# Relationship between Knowledge Management and Social Value among Iranian Nurses

**DOI:** 10.1155/2024/9953915

**Published:** 2024-07-18

**Authors:** Leili Rezaei, Nahid Dehghan Nayeri, Hadis Ashrafizadeh, Fatemeh Hajibabaee, Roohangiz Jamshidi Orak

**Affiliations:** ^1^ School of Nursing and Midwifery Tehran University of Medical Sciences, Tehran, Iran; ^2^ Department of Nursing Management School of Nursing & Midwifery Nursing and Midwifery Care Research Center Tehran University of Medical Sciences, Tehran, Iran; ^3^ School of Nursing Student Research Committee Dezful University of Medical Sciences, Dezful, Iran; ^4^ Nursing and Midwifery Care Research Center School of Nursing and Midwifery Tehran University of Medical Sciences, Tehran, Iran; ^5^ Nursing Care Research Center Iran University of Medical Sciences, Tehran, Iran

## Abstract

**Aim:**

This study was conducted to determine the association between knowledge management and social value among nurses working in hospitals of Tehran University of Medical Sciences.

**Background:**

Knowledge management plays a significant role in healthcare systems. Healthcare providers require knowledge in every aspect of their work, and they must be able to rely on a knowledge management system to access the newest research and practice to ensure the highest quality of care. One of the evident goals of knowledge management is creating value in organizations. Creating value does not necessarily mean creating economic value, but creating social value is a category proposed as a prerequisite for knowledge management.

**Methods:**

This research is a descriptive-analytical study conducted in Tehran in 2021 on two groups of nurses (*N* = 228) selected through a stratified random method. The data collection tools were Choi knowledge management questionnaire and a researcher-made questionnaire on social value validated in Iranian society.

**Results:**

There was a positive and significant correlation between the variables of knowledge management and social value in Bachelor of Science (BSc) nurses (*P* value ≤0.01, *r* = 0.43), and no significant correlation was observed in Master of Science (MSc) nurses (*P* value >0.05, *r* = 0.14).

**Conclusions:**

In the BSc nurses' group, a direct and significant association was found between knowledge management and social value of BSc nurses so that by increasing the score of knowledge management in the nursing community, individuals feel more self-worth, resulting in achieving a favorable level of customer satisfaction. *Implication for Nursing Management*. It is suggested that an accurate program should be designed for all academic levels of nurses in hospitals, the principal elements of creating knowledge and learning and its provision should be assessed, and the necessary measures should be taken.

## 1. Introduction

The necessity for rapid access to information in order to support decision-making in critical conditions in healthcare systems is inevitable. The world has undergone widespread changes during the recent decades culminating in new complications and challenges for all organizations, especially organizations that provide healthcare services. A new field called “knowledge management” has appeared to adapt to these changes [1]. Knowledge management takes a great step towards efficient management by emphasizing learning, organizational thinking, a sense of cooperation, and approach orientation, encouraging individuals to express new ideas, forming an appropriate environment for expressing interests, and identifying individuals' abilities and capabilities and using them in executive affairs and decision-making and assigning them responsibility [2]. By using knowledge discovery in scientific and clinical research, teaching, and other fields extensively to solve complicated problems and improve work efficiency, service provider systems are known as knowledge-based organizations that need skilled and knowledgeable staff [3]. Knowledge discovery, as the first step in the knowledge management process, turns information into knowledge and thereby helps healthcare service providers reduce information overload [4]. Knowledge, as an important resource, should also be managed effectively by the medical staff, particularly nurses, because organizations providing healthcare services need to collect, analyze, and turn data into information and knowledge for decision-making [5]. Having a clear understanding of the information required by nurses to design knowledge management systems is necessary [6]. Some of the positive consequences of knowledge management in the healthcare system are reduced medical errors, improved care quality, improved organizational learning, and increased collaboration, innovation, and reduced costs [7]. Nurses' knowledge management in different studies indicates different results. In Hassanian et al.'s study, knowledge management of the majority of nurses (60.4%) was reported at an average level [8], while in Lee et al.'s study, the mean score of nurses' knowledge management was 3.21 [9], requiring more examinations in the field of knowledge management among nurses. Also, considering the effect of educational levels on knowledge management, it is necessary to compare the knowledge management of nurses with different educational levels [10].

One of the evident goals of knowledge management is creating value in organizations. One of the most general ways to create value is to support efficient and effective decision-making. Creating value does not necessarily mean creating economic value, but creating social value is a category proposed as a prerequisite for knowledge management [11].

In the present organizational atmosphere, “social values” can make anything accessible, and without values, no action works [12]. These values are comprehensible, have an emotional burden, are shared among many individuals and groups, and are used as judgmental norms and a standard for discovering goals and ways to achieve them [13, 14]. Nursing is a profession manifesting its value in the relationship of the individual with the environment and colleagues. This profession provides precious services under challenging conditions to society, while every action contains a variety of values manifesting its actual meaning [15]. Social and moral values include new fields previously mentioned by Florence Nightingale [16]. Nursing social value is a fundamental value in this profession that can foster in the nurse simultaneously with providing nursing care, and this value can be promoted via training so that despite the crises experienced in society, promotes the individuals' perception of the field of nursing and its social value [15]. As a social action, nursing has a variety of values that give it meaning, i.e., these values reflect the practical principles of nursing and build nursing care based on them [15]. Among the most crucial values are social values. The results of a study conducted to determine the social values affecting patient-nurse interactions indicated that the most important social values influencing the patient-nurse relationship included affection or compassion, honesty in words and actions of nurses, and justice and equality in benefitting from medical facilities and nursing services [17]. It is noteworthy that according to Gallup's annual report, nurses have been chosen for the 20th consecutive year by the population of the United States as the specialists who have gained the most trust due to their honesty and ethics, and with 81%, they ranked higher than doctors, teachers, and pharmacists [18]. Moreover, the findings of another study indicate that the value that society places on nursing is positive and is viewed as a profession necessary to improve individuals' health [19].

Despite the numerous advantages of knowledge management, not much research has been conducted on knowledge management and social value in nursing in hospitals, particularly among nurses facing with so much data in various situations. Socialization has not taken place in nursing. A notable point here is that despite the importance of recognizing the variables' positive effects, the resources still have not obtained accurate information regarding the interaction and relationship between these variables in nurses; therefore, by tracking information accurately and recognizing the variables and their role in the nurses' organizational performance exactly, the research team conducted the current research to determine the relationship between knowledge management and social value among nurses working in hospitals of Tehran University of Medical Sciences.

## 2. Methods

### 2.1. Study Design

This research is a descriptive-analytical study conducted in 2020 in all public hospitals of Tehran University of Medical Sciences.

### 2.2. Study Participants

The research population included all BSc (*N* = 4376) and MSc (*N* = 213) nurses working in public hospitals affiliated with Tehran University of Medical Sciences. The sample size was calculated using the pilot study's correlation coefficient (0.26) for type I statistical error to be 0.01 based on the formula (*n*=(*z*/*w*)^2^+3) with ratios (*w*=1/2 ln(1+*r*/(1 − *r*))) [20, 21], and finally, considering 15 probable drops, 112 people were determined as two separate research populations for each of the two groups of MSc and BSc nurses. Nurses were selected using a stratified random sampling method with allocation proportional to the size of the categories in the society so that 14 hospitals affiliated with Tehran University of Medical Sciences were regarded as categories. Based on the number of nurses working in each category, the number of nurses required in each category (hospital) was calculated using mathematical proportionality. After determining the number of nurses in each hospital, the number of nurses that should be placed in the research sample was specified using a table of random numbers. After going to each hospital and providing the random numbers to the nursing office, the researcher received the phone numbers of the sample individuals using the staff list and sent the research questionnaire to them. It should be mentioned that although the sample size was 112 people based on the formula, since the results of the proportionalities resulted in gaining decimal numbers, rounding off the numbers resulted in adding 2 units to the sample size. Thus, the final sample size was obtained at 114 in each group.

### 2.3. Study Tools

The data collection tools in this study consisted of three parts.

The first part was regarding demographic characteristics, including gender, age, marital status, education level, type of employment, service record, service department, and service record in the department.

The second part was Choi knowledge management questionnaire (2005) [22] designed specifically for nurses containing 23 items with a five-point Likert scale: five items regarding knowledge sharing culture, 6 items regarding nursing management system, 4 items regarding creative management leadership, 5 items regarding organizational learning, and 3 items regarding the performance outcomes-based reward system. The options of this scale are completely agree (5), agree (4), no idea (3), disagree (2), and completely disagree (1). The range of attainable scores in this questionnaire is 23–115. Higher scores denote higher levels of knowledge management. The questionnaire's validity and reliability were assessed and approved in Lee et al.'s [9] study, and Cronbach's alpha was reported as 93% [9]. Since Choi knowledge management questionnaire had not been ever translated into Persian, the translation-back-translation process was performed after asking for permission and consent to translate the mentioned questionnaire. After completing the translation process and performing face validity and content validity, the test-retest method was used to determine the knowledge management questionnaire's reliability. For this purpose, according to the opinion of the statistical consultant, the questionnaires were filled out by 20 nurses working in Imam Khomeini Hospital Complex and Shariati Hospital (10 nurses each) selected randomly. After two weeks, the questionnaires were provided to the same nurses again to respond, and the correlation coefficient was then calculated between the two groups of obtained data. The correlation coefficient for the knowledge management questionnaire was reported to be 0.8.

The third part of the social value questionnaire was researcher-made. Despite the existence of a number of tools in this field, none of them had been used specifically in nursing [23, 24]. According to the characteristics and conditions of the nursing profession, a researcher-made tool was designed from a combination of different tools [17, 23, 24]. It was provided to 10 faculty members of the Faculty of Nursing and Midwifery to assess and approve the qualitative and quantitative content validity and face validity. The primary number of questions was 45 questions, and after the faculty members' expert opinions and removing questions with the same concept, 29 questions remained. The content validity index (CVI) and content validity ratio (CVR) of the social value questionnaire in this study were calculated as 0.81 and 0.73, respectively. The test-retest method was used to determine the reliability of the social value questionnaire. For this purpose, according to the statistical consultant's opinion, the questionnaires were completed by 20 nurses working in Imam Khomeini Hospital Complex and Shariati Hospital (10 nurses each) selected randomly. After two weeks, the questionnaires were provided to the same nurses again to respond, and the correlation coefficient was then calculated between the two groups of obtained data. The correlation coefficient for the social value questionnaire was reported to be 0.79. It should be mentioned that these 20 people were excluded from the research sample and the data obtained from them were not used.

### 2.4. Study Processes

Considering the coronavirus disease-2019 (COVID-19) epidemic and its resulting restrictions, data were collected as self-reports by the investigated units, and the questionnaires were sent online. Sampling was performed after confirming the questionnaires' validity and reliability. Because of the restrictions for the continuation of the COVID-19 epidemic, an in-person referral was not possible for the researcher; therefore, online sampling was performed by sending the questionnaire link to the cell phone and email of the selected research unit, which was designed by the researcher in Porsline software (https://lilirezaei.digisurvey.net/40bmd). After filling out and sending the questionnaire by the research units, the data were saved and retrieved in the researcher's email box. The questionnaire was designed in such a way that the research units were able to read, complete, and send the questionnaire as a self-report by spending approximately 10 minutes. They were supposed to answer all questions to submit successfully.

### 2.5. Study Analysis

After entering into SPSS software version 16, the data were statistically analyzed. First, given the participants' responses to the questionnaire questions, the scores obtained by them for the two main variables of the research, i.e., the knowledge management was calculated and classified into the following five scores: very few, few, neutral, high, and very high, and social value was calculated and classified into the following four scores: very low, low, neutral, high, and very high. In order to achieve particular goals and describe the research's main and background variables, descriptive statistics, including frequency distribution tables, central indices such as mean, dispersion indices such as standard deviation, were used for quantitative variables such as age, work experience, knowledge management, and social value, and inferential statistics including the independent *t*-tests, chi-square, analysis of variance (ANOVA), and the nonparametric Mann–Whitney *U* test were used for significant difference of variables between the two groups.

## 3. Results

Based on the research findings, the mean ± SD age of BSc and MSc nurses is 35.89 ± 7.23 and 39.41 ± 7.50, respectively. Furthermore, the mean work experience for BSc nurses was 11.86 ± 7.35 and for MSc nurses was 14.94 ± 7.05. Other demographic information is presented in Table 1.

The research results indicated that the mean ± SD score of knowledge management in BSc nurses was 78.12 ± 16.13 and in MSc nurses was 75.96 ± 19.47. Over one third of the individuals in both investigated groups had a knowledge management score between 79 and 96, which is high. None of the BSc nurses gained a score under 42 and the mean score of this group was higher than that of the MSc group. Although according to the statistical test the existing difference was not significant, it can be said that the two groups had almost the same situation regarding the knowledge management score.

Moreover, the mean ± SD score of social value and the score range in BSc and MSc nurses are 121.15 ± 11.82 (74–145) and 122.18 ± 10.88 (90–144), respectively. Over half of the investigated individuals have gained a very high score (122–145) in evaluating social value. Although given the lowest score and mean score the MSc nurses group is in a better situation regarding social value than the BSc group, the present difference is not to the extent that the statistical test shows it to be significant. Therefore, the two investigated groups are identical regarding the social value score (Table 2). Also, a positive and significant correlation was observed between knowledge management and social value in BSc nurses (*P* value ≤0.01, *r* = 0.43), but there was no significant correlation in MSc nurses (*P* value >0.05, *r* = 0.14) (Table 3).

## 4. Discussion

The present research was carried out to assess the relationship between knowledge management and social value in nurses working in the hospitals of Tehran University of Medical Sciences.

### 4.1. Nurse's Knowledge Management

The results of this research indicated that over one-third of all participants in each group (BSc and MSc nurses) had high knowledge management in the range of 79–96; in other words, they are located in a range indicating that their level of knowledge management is high. In the BSc nurses' group, no nurse was placed in the very low range of knowledge management. Moreover, no significant difference was reported regarding knowledge management between the two groups of BSc and MSc nurses. The high level of knowledge management in nurses causes them not to be anxious in administrating affairs in dealing with unpredicted events. Therefore, there is constantly an answer for each possible question and they solve the patients' problems with their knowledge. The high level of knowledge management in nurses necessitates their better perception of medical issues of the day, and this important issue directs them towards having higher social values. This study was consistent with Jahanbani et al.'s [25] study which reported the knowledge management state as desirable [25]. Despite the limited number of studies investigating the use of knowledge management technology in developing countries, particularly in Iran, studies indicate that the skills and awareness of the use of knowledge management technology are very low among healthcare service providers [26]. In supporting the abovementioned concept, Adane et al. showed in a report on the use of computers and the Internet by nurses in a Nigerian educational hospital that only 43% of the nurses could use knowledge management [27]. Similarly, only 33% of the health specialists in Ethiopia use knowledge management technology for various purposes [26]. In another study, Hosseini-Zahmatkesh et al. [28] evaluated the knowledge management level of nurses at an average level, which was inconsistent with the findings of the current research [29]. The difference between the mentioned study and the current research is that this study has merely investigated the community of BSc nurses and has used a different questionnaire [28]. Ahsan et al. [30] carried out another study to investigate the role of nursing care education based on knowledge management in infection prevention. In this study, the knowledge management level of nurses was assessed based on the pretest and post-test, indicating that after receiving training, the knowledge level of nurses was promoted by 38%. The mean score of knowledge management in the pretest was 56% and in the post-test was 85%. Their study and the current research are different in terms of different knowledge management questionnaires, the different number of samples, and different methods [30]. Since today's successful organizations are organizations that have been able to induce or acquire new knowledge and use it practically in improving and promoting their activities, hospitals should also pay attention to this crucial matter, provide the required context, and encourage nurses to benefit from new and scientific methods to reform their performance.

### 4.2. Nurse's Social Value

The results of this study also indicated that over 50% of the nurses in both groups had a high level of social value. Zarei et al. (2011) reported in their research a high mean score of value perceived by inpatients. These findings reveal a high capacity to promote the value of services provided in hospitals. Guimaraes et al. [15] carried out a qualitative study to identify and perceive social value in the discourse of graduate nursing students and found that social value could be identified and perceived in the discourse of nursing students, being manifested in care pragmatism based on solidarity. As a result, the students acknowledged social value as the main trait in this profession. In the clinical setting, in contact with the patient and his/her family, the students were able to take the social value into consideration using sympathy and consider it necessary for nursing care, recognize patients as human beings, and value them. Students cared about patients not only from a physical-biological viewpoint but also due to their belief in humanity. It means they understood patients as individuals and tried to communicate with them through dialogue [15]. Yang et al. reported that nurses significantly influence the general population [31]. Glerean et al.'s study also displays an image of nursing that is precious to people and is definitely necessary for them [32]. In another study, Vega et al. indicate high levels of trust in nurses and report that 97% of the users welcome nurses in their homes, 76% consider nurses reliable to apply new techniques, and 50% know nurses as reliable to prescribe medication [33], which the results of all these studies are the same as the findings of the current research. However, Pierrotti et al.'s study, although has considered nurses as important as doctors, reported that doctors have more credit than nurses [34]. According to the findings of Girvin et al.'s study, the nursing profession is sometimes not recommended by school vocational counselors or family members since it is not regarded as an ideal career [35]. On the contrary, in Vega et al.'s study, 73.4% of the respondents knew this profession as an acceptable career to be recommended to their loved ones [33]. Some studies indicate that nurses are generally depicted in the newspapers as professionals with a secondary role concerning another profession having low responsibility, independence, or decision-making capacity. It is also recognized as an uninteresting and challenging career, without creativity and responsibility, with few opportunities for growth or promotion, low scientific level, low salary, and low social position [35, 36]. On the contrary, the findings of Vega et al.'s study disagreed with the image of the nursing profession depicted by the media due to injustice regarding the profession's social position or prestige [33]. Some websites belonging to official institutions and healthcare centers suggest the superiority of medicine over other health professions [35]. The results of these studies differ from the findings of the present research, which may be due to different contexts of the nursing profession in different countries, the type of social value recommended in the study, the tools used, and the comparison of nursing with some other professions.

### 4.3. Relationship between Knowledge Management and Social Value

Finally, the results of this study indicated that the scores of knowledge management and social value in the BSc nurses' group had a statistically significant positive association in such a way that as the score of one of the variables increases, the score of another variable also increases, while no such association exists in the MSc nurses group. No study was found that has investigated the association between these variables. Perhaps one of the possible causes of the lack of association between the knowledge management and social value in the MSc nurses' group is that social value is fully formed in these nurses and is not related to other variables including knowledge management. However, Fulk and Yuan et al.'s study indicates that the clarity of individuals' interaction in the organizational social network value helps scholars acquire knowledge-related materials, leading to facilitating interaction with knowledge sources. Hence, the organizational social network value among social media programs is superior to traditional knowledge management systems in eliminating these challenges [37]. According to Ellison et al., organizational social network values culminate in improving knowledge sharing in multinational organizations via raising social capital, supporting relationships, and mutual effects. Organizational social network values make it possible to create knowledge management with various benefits according to the levels of control and interaction [38]. Furthermore, using organizational social network values is positively linked to staff performance [38]. It can be said that one of the obvious goals of knowledge management is to create value in organizations. Knowledge management can pave the way to promote the organization's performance and achieve the sublime goals correctly by integrating the organization's social value in different departments and directly affecting concepts such as customer orientation, organizational learning, leadership, and intelligent management and decision-making, generating new knowledge, turning implicit knowledge into explicit knowledge, and paying attention to individuals' and experts' knowledge. The abovementioned items are among the principal functions of knowledge management that organizations need to achieve their goals. The abovementioned items are among the knowledge management functions that organizations strongly need to achieve their goals.

### 4.4. Limitations

The statistical population of the present research consisted of BSc and MSc nurses working in the hospitals of Tehran University of Medical Sciences; therefore, the results of this study may be generalized to other societies and universities of medical sciences cautiously. Using the questionnaire is considered a limitation in such a way that a questionnaire assesses individuals' attitudes, not the reality, which can be viewed as a limitation. Moreover, when responding, nurses may think that the actual response harms them and respond conservatively, and consequently, the results may be doubted and threatened.

### 4.5. Implication for Nursing Management

It is suggested that an accurate program should be designed for all academic levels of nurses in hospitals, the principal elements of creating knowledge and learning and its provision should be assessed, and the necessary measures should be taken. Moreover, a platform should be formed for the acceptance of using knowledge management on behalf of each organization's staff by revealing the importance of this matter through participation, organizational commitment, extending staff's communication, and using virtual education platforms. In the meantime, simultaneously with creating and developing knowledge management and the affecting factors, the organization's managers should further emphasize the internalization and socialization of knowledge and focus less on its integration, which can promote clients' satisfaction and organizational productivity.

## 5. Conclusions

Based on the results of this study, knowledge management is directly and significantly related to social value in the BSc nurses' group so that by increasing the score of knowledge management in the nursing community, individuals feel more self-worth, resulting in achieving a favorable level of customer satisfaction. Due to large numbers of staff, professors, students, and beneficiaries at various levels, medical sciences and nursing require strong and coherent infrastructural dimensions regarding knowledge management.

## Figures and Tables

**Table 1:** Demographic and clinical characteristics of the nurses participating in the study by academic degree.

Variables		Bachelor nursing		Master sciences nursing		Test statistics	*P* value
		*N*	%	*N*	%		
Gender	Female	94	82.5	88	77.2	*χ* ^2^ = 0.98	0.32
	Male	20	17.5	26	22.8		

Age	20–25	9	7.9	0	0	*T* = −3.60	≤0.01
	26–30	23	20.2	19	16.7		
	31–35	25	21.9	17	14.9		
	36–40	29	25.5	30	26.3		
	41–45	16	14	20	17.5		
	46–50	9	7.9	23	20.2		
	51–55	3	2.6	3	2.6		
	56–58	0	0	2	1.8		

Marital status	Single	38	33.3	40	35.1	*χ* ^2^ = 0.14	0.70
	Married	76	66.7	72	63.2		
	Divorced	0	0	2	1.7		

The university obtained the last degree	Free cities	26	22.8	8	7	*χ* ^2^ = 28.46	≤0.01
	Azad Tehran	13	11.4	12	10.5		
	Azad Research Sciences	0	0	1	0.9		
	Tehran, Beheshti, Iran	26	22.8	60	52.6		
	Rehabilitation	0	0	4	3.5		
	Baqiyatallah	1	0.9	2	1.8		
	Shahed Tehran	2	1.8	1	0.9		
	Government of the cities	46	40.03	26	22.8		

Having a degree other than nursing	Yes	16	14	24	21.1	*χ* ^2^ = 1.94	0.16
	No	98	86	90	78.9		

Another type of educational qualification	Medically related	8	50	9	37.5	*χ* ^2^ = 0.61	0.43
	Nonmedical related	8	50	15	62.5		

Employment status	Permanent	57	50	92	80.8	*χ* ^2^ = 35.74	≤0.01
	Contractual	17	14.9	16	14		
	Corporate	16	14	3	2.6		
	Projective	17	14.9	0	0		
	Temporary	7	6.2	3	2.6		

Work experience	Less than a year	6	5.2	0	0	*T* = −3.22	≤0.01
	1–4	18	15.8	13	11.4		
	5–9	23	20.2	14	12.3		
	10–14	27	23.7	31	27.2		
	15–19	18	15.8	21	18.4		
	20–24	17	14.9	25	21.9		
	25–29	5	4.4	10	8.8		

Ward section	Intensive care	39	34.2	42	36.8	*χ* ^2^ = 22.96	≤0.01
	Emergency	9	7.9	8	7		
	Internal	16	14	14	12.3		
	Surgery	17	15	11	9.6		
	Children	8	7	2	1.8		
	Nursing station	8	7	30	26.3		
	COVID-19	6	5.3	1	0.9		
	Other	11	9.6	6	5.3		

Work experience in the current ward	Less than a year	20	17.5	7	6.1	*T* = −0.57	0.56
	1–4	35	30.7	36	31.6		
	5–9	26	22.8	35	30.7		
	10–14	18	15.8	24	21.1		
	15–19	10	8.8	8	7		
	20–24	5	4.4	4	3.5		

Shift work	Fixed morning	27	23.6	44	38.6	*χ* ^2^ = 3.85	0.14
	Fixed era	4	3.5	2	1.8		
	Fixed night	2	1.8	1	0.9		
	Rotating shift	43	37.7	34	29.8		
	Morning-evening	31	27.2	24	21		
	Evening-night	5	4.4	7	6.1		
	Morning-night	2	1.8	2	1.8		

Employment in another hospital	Yes	11	9.6	9	7.9	*χ* ^2^ = 0.21	0.64
	No	103	90.4	105	92.1		

Total		114	100	114	100		

**Table 2:** Determining the score of knowledge management and social capital according to the level of education of nurses.

Variables		Bachelor nursing		Master sciences nursing		Test statistics	*P* value
		*N*	%	*N*	%		
Knowledge management	23–41 (very few)	0	0	4	3.5	*T* = 0.91	0.36
	42–59 (few)	17	14.9	22	19.3		
	60–78 (moderate)	40	35.1	32	28.1		
	79–96 (high)	45	39.5	39	34.2		
	97–115 (very high)	12	10.5	17	14.9		
	Total	114	100	114	100		

Social value	29–52 (very low)	0	0	0	0	*T* = −0.68	0.49
	53–75 (low)	1	0.9	0	0		
	76–98 (moderate)	3	2.6	3	2.6		
	99–121 (high)	48	42.1	42	36.9		
	122–145 (very high)	62	54.4	69	60.5		
	Total	114	100	114	100		

*Note.* The knowledge management was calculated and classified into five scores as follows: very few, few, moderate, high, and very high. Social value was calculated and classified into five scores as follows: very low, low, moderate, high, and very high.

**Table 3:** Association between two variables in two separate groups.

Variables	Groups			
	Bachelor science group		Master science group	
	*r*	*P* value	*r*	*P* value
Knowledge management and social value	0.43	≤0.01	0.14	≤0.01

## Data Availability

The datasets used and analyzed during the current study are available from the corresponding author on reasonable request.
